# Increased stiffness of median nerve in systemic sclerosis

**DOI:** 10.1186/s12891-017-1793-9

**Published:** 2017-11-07

**Authors:** Ilker Yagci, Ozge Kenis-Coskun, Tugba Ozsoy, Gulsen Ozen, Haner Direskeneli

**Affiliations:** 1 Marmara University Hospital, Physical Medicine and Rehabilitation Department, Istanbul, Turkey; 2 Kartal Research and Training Hospital, Physical Medicine and Rehabilitation Department, Şemsi Denizer Caddesi, 34865 Kartal, Istanbul, Turkey; 3 Marmara University Hospital, Rheumatology Department, Istanbul, Turkey

**Keywords:** Carpal tunnel syndrome, Median nerve, Sonoelastography, Systemic sclerosis

## Abstract

**Background:**

Systemic sclerosis can affect peripheral nerves, but the extent and the nature of this involvement are not well defined. The aim of this study is to compare the sonoelastrographic measurements of median nerves in systemic sclerosis (SSC), idiopathic carpal tunnel syndrome (CTS) and healthy individuals.

**Methods:**

The clinical, electrophysiological and ultrasonographic assessments were done. Patients with SSC and CTS were assessed with nerve conduction studies. The measurements of cross sectional areas (CSA) were performed at psiform and forearm level from axial US images. The elastic ratio is the ratio of strain distribution in two selected region of interests (ROI) done via comparing the median nerve to flexor digitorum superfcialis tendon. The ROIs were fixed to 2 mm.

**Results:**

The study was completed with 47 hands of 24 patients with SSC, 53 hands of 27 patients with CTS and 38 hands of health controls. The CSA of CTS group was significantly higher than systemic sclerosis and control groups. The elastic ratio at psiform level and forearm levels of systemic sclerosis group were significantly higher than the CTS and control groups.

**Conclusion:**

Median nerves lose the elasticity while the CSA’s are in the normal range in patients with SSC. These results suggested that the increased peripheral nerve involvement in SSC is about the increased stiffness of the nerves.

## Background

Systemic sclerosis (SSC) is an autoimmune disease of unknown etiology that causes thickening in the connective tissues [1]. The prognosis is mainly affected by the involvement of visceral organs. However, the involvement of the musculoskeletal system can take a major part in the disability caused by the disease [2]. SSC can affect peripheral nervous system, however, the importance and frequency of neurologic involvement in SSC has been debated. In a systematic review trigeminal neuropathy (16.5%), peripheral sensorimotor polyneuropathy (14.3%), and carpal tunnel syndrome (6.6%) were the most frequent forms of peripheral nervous system involvement in SSC [3].

Carpal tunnel syndrome (CTS) is the entrapment of median nerve under the flexor retinaculum. [4]. It has been shown that median nerve conduction is affected in SSC, however surgical treatment does not improve symptoms in patients with SSC. It is suggested that compression of the nerve is not the main cause for symptoms [5] and whereas a peripheral nerve involvement can be due to the compression of single nerve fascicles by increased connective tissue of the endo- and perineurium or microangiopathy of the vasa nervorum [6, 7]. Moreover, electrophysiologic or sonographic changes can be seen in median nerve in patients with SSC, without the symptoms of CTS [8]. On the other hand, CTS is a clinical diagnosis, the electrodiagnostic studies are performed to solidify the diagnosis and to document the extent of involvement [9]. A timely diagnosis is warranted to prevent permanent nerve damage and functional loss [10].

Recently, ultrasonography investigation of the median nerve has also been used to visualize median nerve in carpal tunnel. In ultrasonography, an increase in median nerve cross-sectional area and decreased echogenicity due to neural edema within the carpal tunnel are typical findings [11]. Sonoelastography is another emerging technique that evaluates the elasticity of the tissues. It has previously been used to document decreased skin elasticity in patients with SSC [12]. It has also been recently used in the imaging of median nerve and shown to have a role in increasing the diagnostic accuracy of carpal tunnel syndrome via ultrasound [13]. Median nerve elasticity is affected by the presence of carpal tunnel syndrome but the reports about the stiffness of median nerve are conflicting [14, 15]. As a result, there are many questions about the involvement of median nerve in SSC, and how it differs from patients with carpal tunnel syndrome and healthy individuals.

## Method

The major aims of this study were to detect the amount of clinical and electrophysiological peripheral neuropathies in patients with SSC and compare sonographic and elastographic findings of patients with SSC and CTS with healthy individuals defining the differences in elasticity in median nerve in these patients.

### Patients and controls

The study was conducted between May 2014–May 2015. Patients with SSC were recruited from Rheumatology Outpatient Clinics and the patients with idiopathic CTS were recruited from Physical Medicine and Rehabilitation Outpatient Clinic of Marmara University Hospital. The inclusion criteria of SSC were being 18–65 years old female diagnosed as SSC according to the American Collage of Rheumatology 2013 criteria [16]. The inclusion criteria of CTS were female patients between the ages of 18–65 years who have been diagnosed with CTS according to electrodiagnostic and clinical findings. The exclusion criteria were history of previous carpal tunnel surgery or injection or physical therapy in the last 3 months, and any etiologies pointing out a secondary facilitator for CTS like hypothyroidism, pregnancy and diabetes mellitus. Age-matched controls are also recruited from healthy volunteers without a history of any chronic musculoskeletal or rheumatologic disease and a previous history of carpal tunnel symptoms. All the participants have read and signed an informed consent.

All clinical evaluations were performed by the same physician (GO) with a standardized patient assessment form. Neurological examination and electrodiagnostic testing were performed by the second physician (OKC) with standard clinical assessment including muscle strength, sensory testing, knee and ankle stretch reflexes, Tinnel’s sign, Phalen’s test. The sonographic and elastosonographic measurements were performed a by third physician (IY) who was experienced in musculoskeletal ultrasound. These researchers were unaware of each other’s results. The results were collected and analyzed by another researcher (TO).

### Nerve conduction studies

Patients with SSC have undergone a nerve conduction study (NCS) with polyneuropathy protocol which consisted of median and ulnar nerve sensory and motor NCS’s, tibial and peroneal nerve motor NCS’s and sural nerve sensory NCS. Patients with idiopathic CTS were assessed with CTS protocol that consisted of median and ulnar nerve sensory and motor NCS’s and comparative studies. NCS were performed with Medtronic-Keypoint (Denmark, 2007) device by the same physician. All studies were done under standard room temperature of 25 °C. Hand temperature was maintained at 32 °C or greater. The hand temperatures were assessed by device’s thermometer. Median, ulnar and tibial motor nerve proximal and distal latencies, motor nerve conduction velocities, compound muscle action potential amplitudes were measured. Median, ulnar and sural sensory NCS were recorded with standardized methods. For all sensory NCS, distal latency, sensory nerve action potential amplitude, and sensory nerve conduction velocity were recorded. Electrophysiological diagnosis of any neuropathy was obtained according to normative values of our laboratory and an abnormality was defined according to American Association Of Neuromuscular and Electrodiagnostic Medicine.

### Sonoelastography

Sonographic assessments of the median nerve were performed with Esaote Mylab 60 Ultrasound device (Geneva, Italy) and 6–18 Mhz linear transducer. Patients were seated facing the sonographer with forearm lying on the patient’s knee. The elbow was in flexion; the wrist in supination and the fingers were relaxed. The measurements of cross-sectional areas were performed at psiform and forearm level from axial US images. The measurements were obtained from inner of hyperechogenic rim of the median nerve. The measurements were performed and the mean values were recorded.

**Fig. 1:**
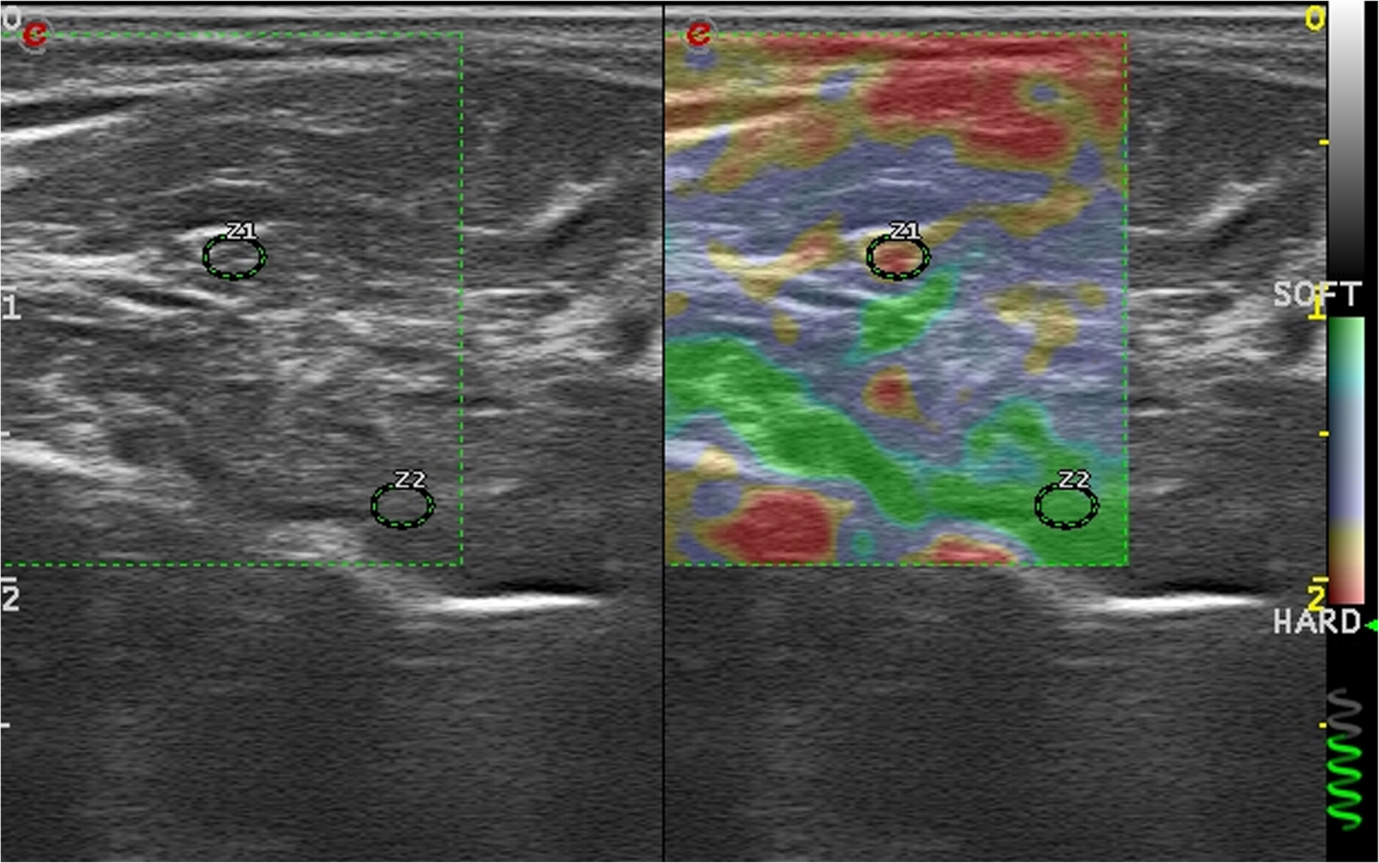
Sonoelastographic measurement of median nerve at forearm level

The sonoelastrography was performed with the same transducer and by the same sonographer. Twin view was used while B mod was on the left and the elastography view was at the right side of the screen. The median nerve was visualized in axial view and kept in center of the screen. The cross-sectional area (CSA) of the median nerve was recorded. The sonographer made seven to ten slight compressions periodically until the device determined that the proper elastographic view was obtained. It was important to do slight compressions in vertical plane and the screen was frozen at the decompression phase. According to the color box; green color represents the minimal and red color represents the maximal stiffness. The elastic ratio is the ratio of strain distribution in two selected region of interests (ROI). A round ROI was positioned in the center median nerve, while a same size of ROI was positioned in a homogeneously soft anatomical structure, which was considered as internal control. Second ROI was positioned on the flexor carpi radialis for the wrist level and flexor digitorum superficialis for the forearm level. The ROIs diameters were fixed to 2 mm. By using this method, a higher elastic ratio reflects a lower elasticity of median nerve (Fig. 1). The measurements have been repeated three times and the median value of these measurements were used in analyses.

### Statistical analyses

The data of the study was collected by TO and the statistical analyses were performed with Statistical Package for the Social Science Program (SPSS Version 10.0). The main characteristics of patients were evaluated with descriptive studies. Comparison of the mean values of NCS parameters was performed with ANOVA and categorical values were analyzed with chi-square tests. The correlation of sonographic, elastographic, electrophysiological and clinical data was performed with Pearson’s correlation test. *P* values lower than 0.05 was accepted as statistically significant.

## Results

Initially, 29 patients with SSC, 27 patients with CTS and 20 healthy controls were enrolled in the study. NCS’s could not be completed in five patients with SSC because of digital ulcers or severe contractures in hand joints. One patient in healthy subjects was excluded because of hereditary sensorimotor polyneuropathy according to NCS. Bifid median nerves were found unilaterally in one patient in SSC and CTS group. The study was completed with 47 hands of 24 patients with SSC, 53 hands of patients with CTS and 38 hands of health controls.

There were no significant differences between the groups according to age (×2:0.13, *p* = 0.13) and gender (F/M: 1.95; *p* = 0.15). For SSC group, disease duration mean was 5.7 ± 1,01 years. Modified Rodnan skin score mean was 11.5 ± 7.4. Raynaud phenomenon, renal crisis, and digital ulcers were present in 21, 1 and 12 patients respectively. Electrodiagnostic testing of SSC group resulted as 17 normal, 4 had carpal tunnel syndrome and 3 had polyneuropathy.

The CSA of CTS group was significantly higher than the control and SSC groups (*p* = 0.001 and *p* = 0.004 respectively). There was no significance between SSC and control group. The elastic ratio at psiform level and forearm levels of SSC group were significantly higher than the control and CTS groups (*p* = 0.001, *P* = 0,04 respectively). There was no statistical difference between CTS and control groups. The sonographic measurements of three groups are listed in Table 1.

**Table 1:** Analysis of sonographic and elastographic data

	Group	Mean	Std. Deviation	ANOVA	
Median nerve cross sectional area at psiform level	Control	9.13	2.18	F: 11.15 *p* = 0.001	Control/systemic sclerosis *p* = 0.325 Control/CTS *p* = 0.001 CTS/systemic sclerosis *p* = 0.004
	Systemic sclerosis	10.27	3.36		
	CTS	12.64	4.61		
Elastic ratio at psiform level	Control	1.63	1.15	F: 7.09 *p* = 0.001	Control/systemic sclerosis *p* = 0.001 Control/CTS *p* = 0.27 CTS/systemic sclerosis *p* = 0.04
	Systemic sclerosis	3.02	2.56		
	CTS	2.19	0.93		
Elastic ratio at forearm level	Control	1.77	1.21	F: 9.93 *p* = 0.001	Control/systemic sclerosis *p* = 0.001 Control/CTS *p* = 0.24 CTS/systemic sclerosis *p* = 0.008
	Systemic sclerosis	3.21	1.81		
	CTS	2.29	1.39		

In correlation analyses forearm elasticity of median nerve was inversely correlated with ulnar motor nerve conduction velocity (r:-0,474 *p* = 0,02), psiform level elasticity of median nerve was inversely correlated with median motor nerve distal latency (r:-0,436 *p* = 0,04) and ulnar motor nerve compound muscle action potential amplitude (r:-0,507 *p* = 0,01). There were no other correlations between nerve elasticity and other electrodiagnostic parameters. There was no correlation between modified Rodnan skin score and other parameters.

## Discussion

The prevalence of peripheral neuropathy has not been well defined in patients with SSC. In a review about nervous system involvement in patients with SSC among total of 61 studies and case reports, 442 peripheral nerve system involvements have been found in total of 1628 patients. The overall reported prevalence for CTS was 6.56% and 14% for PNP [3]. In our study, the percent of patients who had electrodiagnostic CTS was 16% and polyneuropathy was % 12. These results suggested high rates of peripheral neuropathy in patients with SSC.

Previous studies that visualized the median nerve in SSC were not about the elasticity of the nerve. The median nerve area, diameter and flattening ratio were significantly affected in patients with SSC [8] and the ratio between the fascicular and non-fascicular structure of the median nerve was decreased [17]. This is the first study to actually document an increase in the stiffness of the median nerve in patients with SSC. In their study, Bandinelli et al. have concluded that the involvement of median nerve is not due to the pathologies involving the surrounding tissue but rather due to the compromised microvascularision of the median nerve itself [8]. Our study can be added to this hypothesis and claim that the involvement of the connective tissue of the nerve itself also plays a role in patients with SSC, suggesting a multisystem pathophysiology. We documented that the stiffness in SSC was even higher than patients with CTS. Despite stiffness was increased in patients with CTS there was no statistical significance between CTS and control group. There are contradictory results in about median nerve elasticity in CTS in the literature. One previous study shows a decrease in median nerve stiffness. That study was done in pregnant women, who usually have CTS due to edema around the median nerve, caused by an overall increase in body fluid content during pregnancy [15]. However, another study which included 41 CTS patients showed an increase in the stiffness of median nerve, which suggested the fibrosis and scarring in median nerve might have influenced this result that fibrosis and scarring are present in median nerve [14]. In CTS, it is thought that as the compression progresses, the edema and defects in microcirculation affect epineurium and can end up with fibrosis, therefore the stiffness is found to be increased in that study [4]. For our study, the CTS group included commonly mild to moderate cases. We thought that the stiffness can be increased in severe cases and a statistical significance could have been found if there were more severe CTS cases. In further studies, patient groups must be formed according to these pathophysiological properties.

In correlation analysis, we observed a statistical correlation between ulnar nerve conduction studies and nerve elasticity. These results suggested that the elasticity of median nerve decreased in patients with generalized peripheral nerve involvement. We did not test the ulnar nerve elasticity in the study but it is possible that there was also increased stiffness in ulnar nerve in patients with SSC. In addition to this median nerve latency increases with increased stiffness, showing that nerve conduction suffers when the elasticity of neural tissue decreases. No correlation between median nerve motor compound action potential amplitudes may indicate that myelin tissue suffers more than the axon itself, which also correlates with the hypothesis of connective tissue involvement. However, the clinical and demographic features did not correlate with stiffness. A previous study has also failed to show that clinical parameters of scleroderma like Rodnan score have an influence on median nerve [8]. The lack of any correlations between clinical parameters and median nerve involvement can be due to the limited number of patients that has been involved in these studies. Further studies need to be conducted to evaluate what affects median nerve elasticity in SSC, and if elasticity is involved in other peripheral neuropathies.

## Conclusion

This the first study that demonstrates increased stiffness of median nerve in both wrist and forearm levels. The median nerve stiffness was also significantly higher than patients with CTS. Further studies with higher patient counts and also assessing elasticity of other nerves are needed.

## Abbreviations

CSA: Cross-sectional area
CTS: Carpal tunnel syndrome
NCS: Nerve conduction study
ROI: Region of interest
SSC: Systemic sclerosis
